# Epidermal growth factor receptor-targeted near-infrared probe cetuximab-IRDye800CW enables stable and tumor-specific fluorescence imaging in colorectal cancer models

**DOI:** 10.1117/1.JBO.30.12.126002

**Published:** 2025-12-03

**Authors:** Yong Huang, Xianhu Zhang, Yongjun Jiang, Daorong Wang

**Affiliations:** a Northern Jiangsu People’s Hospital Affiliated to Yangzhou University, Yangzhou, China; b Northern Jiangsu People’s Hospital, Yangzhou, China; c Yangzhou Key Laboratory of Basic and Clinical Transformation of Digestive and Metabolic Diseases, Yangzhou, China

**Keywords:** colorectal cancer, fluorescence-guided surgery, cetuximab, IRDye800CW, tumor resection

## Abstract

**Significance:**

Colorectal cancer (CRC) remains a major cause of cancer-related mortality, with incomplete resection contributing to recurrence and poor survival. Improving intraoperative margin detection is critical to enhance surgical precision and patient outcomes.

**Aim:**

We aim to evaluate the tumor-targeting specificity of cetuximab-IRDye800CW (Cet-IRDye800CW), an epidermal growth factor receptor (EGFR)-specific near-infrared probe, for fluorescence-guided surgery in CRC models.

**Approach:**

Cet-IRDye800CW and IgG-IRDye800CW controls were synthesized and tested in LS174T and SW948 xenografts as well as patient-derived xenografts (PDXs). Mice received 200  μg of the probe intravenously and underwent daily fluorescence imaging for 10 days. Tumor-to-background ratio (TBR) and mean fluorescence intensity (MFI) were quantified, and EGFR expression was analyzed by Western blot, reverse transcription quantitative polymerase chain reaction, and immunohistochemistry.

**Results:**

Cetuximab-IRDye800CW significantly enhanced tumor fluorescence compared with controls in all models (p<0.05). TBR increased progressively and peaked on day 10 (LS174T: 25.6±2.94; SW948: 21.6±1.71), whereas background MFI declined, resulting in improved tumor contrast. Tumor-specific fluorescence was detectable from day 1 and intensified over time. Similar findings were observed in PDX models, consistent with high EGFR expression.

**Conclusion:**

Cetuximab-IRDye800CW provides stable, tumor-specific, high-contrast fluorescence imaging in EGFR-high CRC models. These findings validate its molecular targeting capability and support its translational potential for fluorescence-guided CRC surgery.

## 1 Introduction

Colorectal cancer (CRC) is one of the leading causes of cancer-related morbidity and mortality worldwide, and complete surgical resection remains the cornerstone of curative treatment. However, achieving negative margins is particularly challenging when tumors are located in anatomically complex regions or lack clear boundaries with surrounding normal tissue. Incomplete resection contributes to high recurrence rates and poor survival in patients with advanced CRC,^1^ underscoring the urgent need for strategies that improve intraoperative visualization of tumor margins.

Fluorescence-guided surgery (FGS) has emerged as a promising approach to enhance the precision of oncologic resections. Using fluorescent probes that preferentially accumulate in tumor tissue, FGS provides real-time delineation of tumor boundaries, thereby improving resection accuracy while minimizing damage to adjacent healthy structures.^2^^,^^3^ Conventional fluorophores such as indocyanine green (ICG) lack tumor specificity and often yield suboptimal contrast. By contrast, cetuximab-IRDye800CW (Cet-IRDye800CW), a conjugate of the anti-epidermal growth factor receptor (EGFR) monoclonal antibody cetuximab and the near-infrared dye IRDye800CW, combines molecular specificity with favorable optical properties, offering selective tumor visualization and deep tissue penetration.^4^^–^^7^

In recent years, antibody–dye conjugates with molecular specificity for tumor biomarkers have become a major focus of FGS research beyond conventional nonspecific probes such as ICG. Although ICG-guided imaging shows promise for detecting hepatic and peritoneal metastases of CRC, its nonspecific accumulation limits tumor-to-background contrast.^8^ Consequently, molecularly targeted probes are being actively explored across various tumor types to improve intraoperative visualization. For example, fluorescence imaging has been shown to refine tumor delineation and enhance resection accuracy in glioblastoma surgery.^9^ Among these, cetuximab-IRDye800CW (Cet-IRDye800CW) has gained attention for its excellent near-infrared penetration, high tumor-to-background contrast, and selective tumor accumulation. Multiple clinical studies in non-CRC tumors have confirmed its safety and feasibility. In a phase I dose-escalation trial of head-and-neck squamous cell carcinoma (n=12), patients received 2.5, 25, or $62.5\;\mathrm{mg}/m^{2}$ with no ≥ grade 2 adverse events.^10^ Another study showed that the mean tumor-to-background ratio (TBR) increased with dose (3.6 at 50 mg versus 4.3 at 100 mg) and was significantly higher in contrast-enhancing tumors than in nonenhancing ones (4.0±0.5 versus 1.2±0.3; p=0.02), confirming the feasibility of EGFR-targeted imaging.^11^ Furthermore, ongoing or completed clinical trials have evaluated Cet-IRDye800CW in various malignancies, including head and neck cancer (NCT01987375), malignant glioma (NCT02855086), and pancreatic cancer (NCT02736578), highlighting its translational potential for perioperative tumor visualization.

Although cetuximab-IRDye800CW has been investigated in other malignancies, its performance characteristics in colorectal cancer remain largely undefined—particularly the longitudinal stability of fluorescence signal, the relationship between signal intensity and EGFR expression, and the translational relevance for intraoperative decision-making. Moreover, no study has systematically evaluated both open- and closed-field imaging across cell line-derived and patient-derived xenograft models, which better reflect real surgical conditions and inter-patient heterogeneity. Therefore, this study aimed to comprehensively characterize fluorescence kinetics, background clearance, and EGFR-dependent tumor uptake in colorectal cancer models to establish a solid preclinical foundation for colorectal-specific clinical translation of cetuximab-IRDye800CW.

## 2 Methods

### 2.1 Reagents

Cetuximab (MCE, Monmouth Junction, New Jersey, United States), a chimeric monoclonal antibody targeting the EGFR, was conjugated with the near-infrared fluorescent dye IRDye800CW (IRDye800CW-N-hydroxysuccinimide ester, LI-COR Biosciences, Lincoln, Nebraska, United States) under defined reaction conditions. Cetuximab was diluted to 1  mg/mL in phosphate-buffered saline (PBS, pH 7.4). IRDye800CW was dissolved in anhydrous DMSO to prepare a 10 mM stock solution and then added dropwise to the antibody solution at a molar ratio of 2.3: 1 (dye: protein) with gentle stirring. The reaction was allowed to proceed for 2 h at 20°C in the dark to permit covalent coupling between the NHS-ester group of the dye and the primary amines of the antibody. After incubation, the mixture was purified through centrifugal ultrafiltration columns (Amicon Ultra-15, 10 kDa cut-off, Millipore, Burlington, Massachusetts, United States) and centrifuged at 4000×g for 15 min to remove unreacted free dye. The retentate was washed twice with PBS, concentrated to 2  mg/mL protein, aliquoted, and stored at 4°C protected from light.

Quality-control analyses were performed to verify the conjugation. The dye-to-protein ratio [degree of labeling (DOL)] was calculated from absorbance at 280 and 774 nm and averaged ≈2.3:1. Structural integrity and purity were confirmed by sodium dodecyl sulfate–polyacrylamide gel electrophoresis (SDS-PAGE) and high-performance liquid chromatography (HPLC); sterility and pH were assessed before *in vivo* administration, and EGFR-binding activity was validated *in vitro*.

Protein-A–purified IgG was conjugated with IRDye800CW using the same procedure described above to generate IgG-IRDye800CW, which served as a nonspecific negative control fluorescent probe in all *in vivo* imaging experiments.

### 2.2 Sample Size and Power Calculation

The primary endpoint for sample size planning was the peak TBR within days 1 to 10 postinjection, compared between cetuximab-IRDye800CW and IgG-IRDye800CW groups at the pre-specified peak day. Based on pilot data from our laboratory (LS174T xenografts) showing a mean TBR of 3.2±0.9 for cetuximab-IRDye800CW versus 1.6±0.5 for IgG-IRDye800CW, the standardized effect size (Cohen’s d) was estimated as *(3.2 to 1.6)/SD_pooled* ≈2.2. Assuming a two-tailed two-sample t-test (α=0.05) and 80% power, the required sample size was n=4 per group. To account for potential attrition (anesthesia or imaging-related loss) and to preserve power across cell-line and patient-derived xenograft (PDX) cohorts, we enrolled n=5 mice per group for each cohort.

Longitudinal mean fluorescence intensity (MFI) across 10 daily measurements was analyzed as a secondary endpoint using a linear mixed-effects model (random intercept for mouse; fixed effects: treatment, day, and treatment × day). Although the sample size was driven by the primary endpoint, simulations (1000 runs; intra-mouse correlation ρ≈0.5) indicated power ≥80% to detect a treatment × day interaction corresponding to a medium effect (standardized f≈0.25) with n=5 per group and 10 time points. All calculations were performed in G*Power 3.1 (for t-tests) and verified by custom R scripts (for mixed-model simulations).

### 2.3 Cell Lines and Animal Models

The human colorectal adenocarcinoma cell lines LS174T (KRAS mutant) and SW948 (KRAS wild-type) were cultured in RPMI 1640 medium (Eallbio, Beijing, China) supplemented with 10% fetal bovine serum and 1% Plasmocin (InvivoGen, San Diego, California, United States) at 37°C in a 5% ${\mathrm{CO}}_{2}$ incubator. When cells reached 70% to 90% confluency, they were harvested and counted using a hemocytometer. A total of $1\times 10^{6}\text{ cells}$ were resuspended in 200  μL PBS. Female BALB/c nude mice (4 to 5 weeks old) were randomly divided into two groups (n=10 per group) and subcutaneously inoculated in the left axillary region to establish xenograft tumors. After the tumors reached ∼100 to $150\;mm^{3}$ (around 3 weeks postinoculation), randomization was again applied. Mice bearing LS174T xenografts were randomized into two subgroups: an experimental group and a control group (n=5 per group). Similarly, mice bearing SW948 xenografts were randomized into two subgroups (n=5 per group). The experimental groups received a tail vein injection of 200  μg cetuximab-IRDye800CW, whereas the control groups received 200  μg IgG-IRDye800CW via the same route.

### 2.4 Human PDX Models

Fresh rectal cancer specimens were obtained within 30 min after surgical resection, cut into ∼0.5× $0.5\times 0.5\;cm^{3}$ fragments, and subcutaneously implanted into nude mice. Tumors were serially passaged to the third generation (P3) before use. Six PDX models with confirmed high EGFR expression were established and characterized by Western blotting. For each PDX model, 10 mice were generated through subcutaneous implantation of tumor fragments. The PDX-bearing nude mice were randomly divided into an experimental group (n=5) and a control group (n=5). The experimental group received 200  μg of cetuximab-IRDye800CW via tail vein injection, whereas the control group received 200  μg of IgG-IRDye800CW through the same route. To ensure reproducibility, the experiment was independently repeated in another cohort of PDX-bearing mice (n=3 per group).

**Fig. 1:**
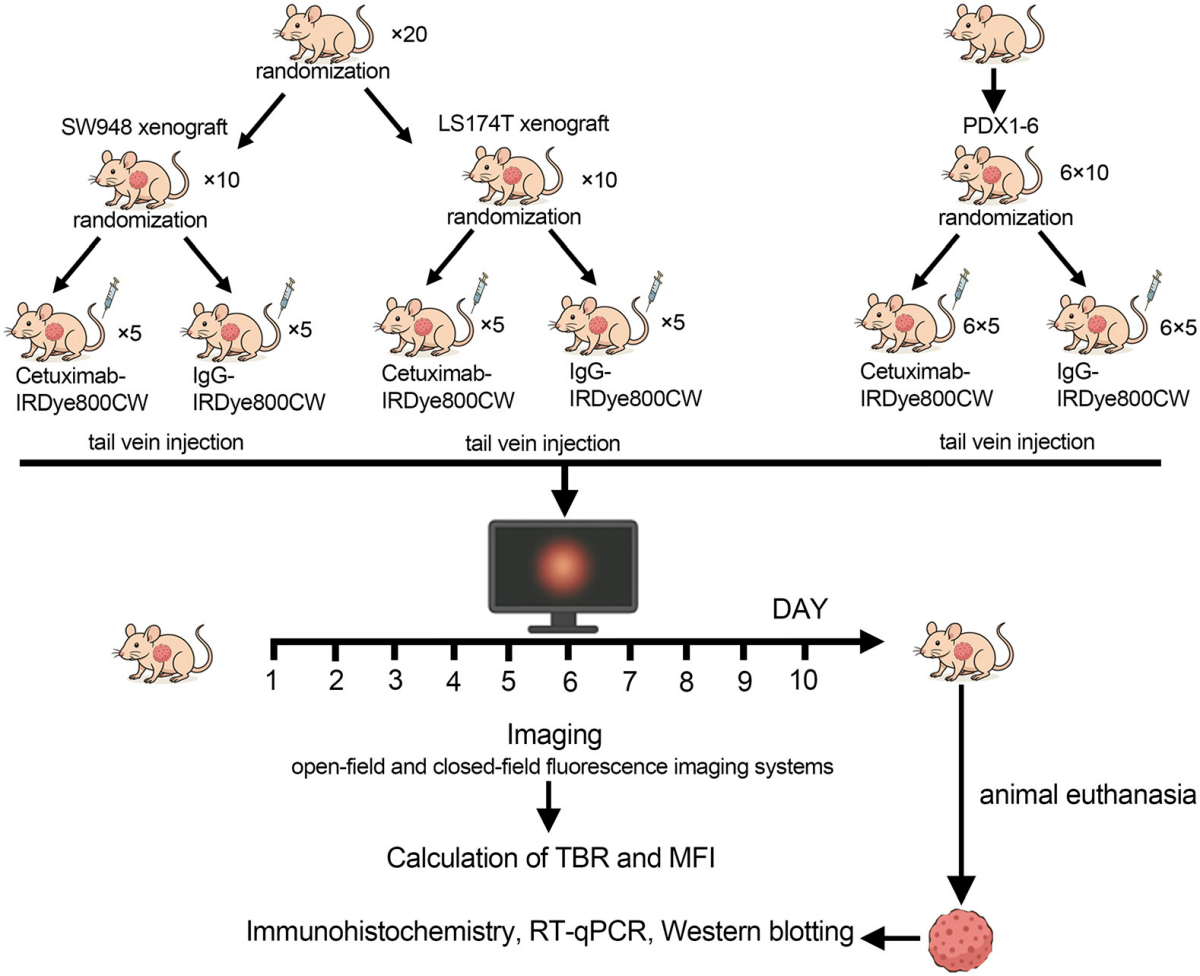
Schematic overview of the experimental design and animal grouping. Female BALB/c nude mice bearing LS174T, SW948, or EGFR-high patient-derived xenograft (PDX) tumors were randomized into two groups (n=5 per group) receiving cetuximab-IRDye800CW (200  μg) or IgG-IRDye800CW (200  μg) via tail vein injection. Fluorescence imaging was performed daily from day 1 to 10 using open-field (LUNA) and closed-field (Pearl Impulse) systems under 775-nm excitation and 795-nm emission. Tumor-to-background ratio (TBR) and mean fluorescence intensity (MFI) were quantified, followed by *ex vivo* imaging and EGFR validation (Western blot, RT-qPCR, IHC).

A schematic overview of the animal grouping and intervention workflow is presented in Fig. 1. All animal experiments were approved by the Animal Experimentation Committee of Yangzhou University and conducted in accordance with institutional ethical guidelines.

### 2.5 *In Vivo* Fluorescence Imaging

To comprehensively evaluate tracer biodistribution and imaging performance, both open-field and closed-field fluorescence imaging systems were employed. Open-field imaging was used to simulate the intraoperative surgical environment and assess the feasibility of fluorescence visualization under ambient light conditions, whereas closed-field imaging was performed under standardized, light-isolated conditions to obtain quantitative fluorescence data.

Mice were imaged daily for 10 consecutive days using both systems. Open-field imaging was performed using the LUNA fluorescence system (Nuliferay, Nanjing, China). Anesthetized mice were positioned 15 cm from the imaging head, and images were acquired with a 10-s exposure under 800 nm excitation. Closed-field imaging was conducted using the Pearl Impulse system (LI-COR Biosciences, Lincoln, Nebraska, United States). Mice were anesthetized and placed in the imaging tray, and images were captured on the 800 nm channel under uniform illumination to minimize background variability. The fluorescent probe used in this study was IRDye800CW (LI-COR Biosciences, Lincoln, Nebraska, United States), with an excitation wavelength of 775 nm and an emission wavelength of 795 nm. On day 10, mice were euthanized, tumors were excised, and the wound beds were re-imaged using both systems. To assess detection sensitivity, ∼1  mg tumor fragments were reinserted into the wound bed and re-imaged.

Quantitative analysis was performed using Image Studio (LI-COR) for closed-field data and SPY-Q (Novadaq, Mississauga, Canada) for open-field data. Regions of interest (ROIs) were manually drawn over the tumor and adjacent normal tissue. Mean fluorescence intensity (MFI) was recorded for each ROI, and the TBR was calculated as: $\mathrm{TBR}={\mathrm{MFI}}_{\text{tumor}}/{\mathrm{MFI}}_{\text{background}}$. TBR values and MFI kinetics were used to compare imaging performance between cetuximab-IRDye800CW and IgG-IRDye800CW groups.

### 2.6 Western Blotting

Cells were lysed in RIPA buffer (Solarbio, Beijing, China) on ice, and lysates were centrifuged at 14,000 rpm for 15 min at 4°C. Protein concentration was determined by BCA assay (Yeasen, Shanghai, China). Equal amounts of protein were resolved by SDS-PAGE and transferred to PVDF membranes (Immobilon-P, Merck Millipore, Burlington, Massachusetts, United States). After blocking with 5% milk, membranes were incubated with primary antibodies (anti-EGFR, 1:8000; glyceraldehyde-3-phosphate dehydrogenase (GAPDH), 1:8000; Abclonal, Woburn, Massachusetts, United States) overnight at 4°C, followed by secondary antibody (goat anti-rabbit IgG, 1:8000; Abclonal) for 1.5 h at room temperature. Protein bands were visualized using ECL reagent (Vazyme, Nanjing, China) and quantified with ImageJ. Experiments were performed in triplicate.

### 2.7 RT-qPCR

Total RNA was extracted using Trizol (Yeasen, Shanghai, China) and reverse-transcribed to cDNA (Yeasen, Shanghai, China). qPCR was performed with SYBR Green PCR kit (Vazyme, Nanjing, China) under the following conditions: 95°C for 5 min, then 40 cycles of 95°C for 5 s and 60°C for 30 s. Primers (Gene Pharma, Suzhou, China) were: EGFR forward 5′-AGGCACGAGTAACAAGCTCAC-3′, reverse 5′-ATGAGGACATAACCAGCCACC-3′; GAPDH forward 5′-GGAGCGAGATCCCTCCAAAAT-3′, and reverse 5′-GGCTGTTGTCATACTTCTCATGG-3′. Relative gene expression was calculated using the 2^−^ΔΔCt method, normalized to GAPDH.

### 2.8 Immunohistochemistry

Tumor samples were fixed in formalin, embedded in paraffin, and sectioned. Sections underwent deparaffinization, antigen retrieval, peroxidase blocking, and goat serum blocking (OriGene, Rockville, Maryland, United States). Primary anti-EGFR antibody (1:50, Abclonal) was applied overnight at 4°C, followed by incubation with secondary antibody (OriGene) for 25 min at 37°C. DAB substrate (OriGene) was used for visualization, and sections were counterstained with hematoxylin (Beyotime, Shanghai, China). Slides were independently evaluated by two blinded pathologists, and results were quantified.

### 2.9 Statistical Analysis

Fluorescence intensity (MFI) and TBR were expressed as mean ± *SD* and compared between groups using two-tailed unpaired Student’s t-test or one-way ANOVA as appropriate. A p-value<0.05 was considered statistically significant.

## 3 Results

**Fig. 2:**
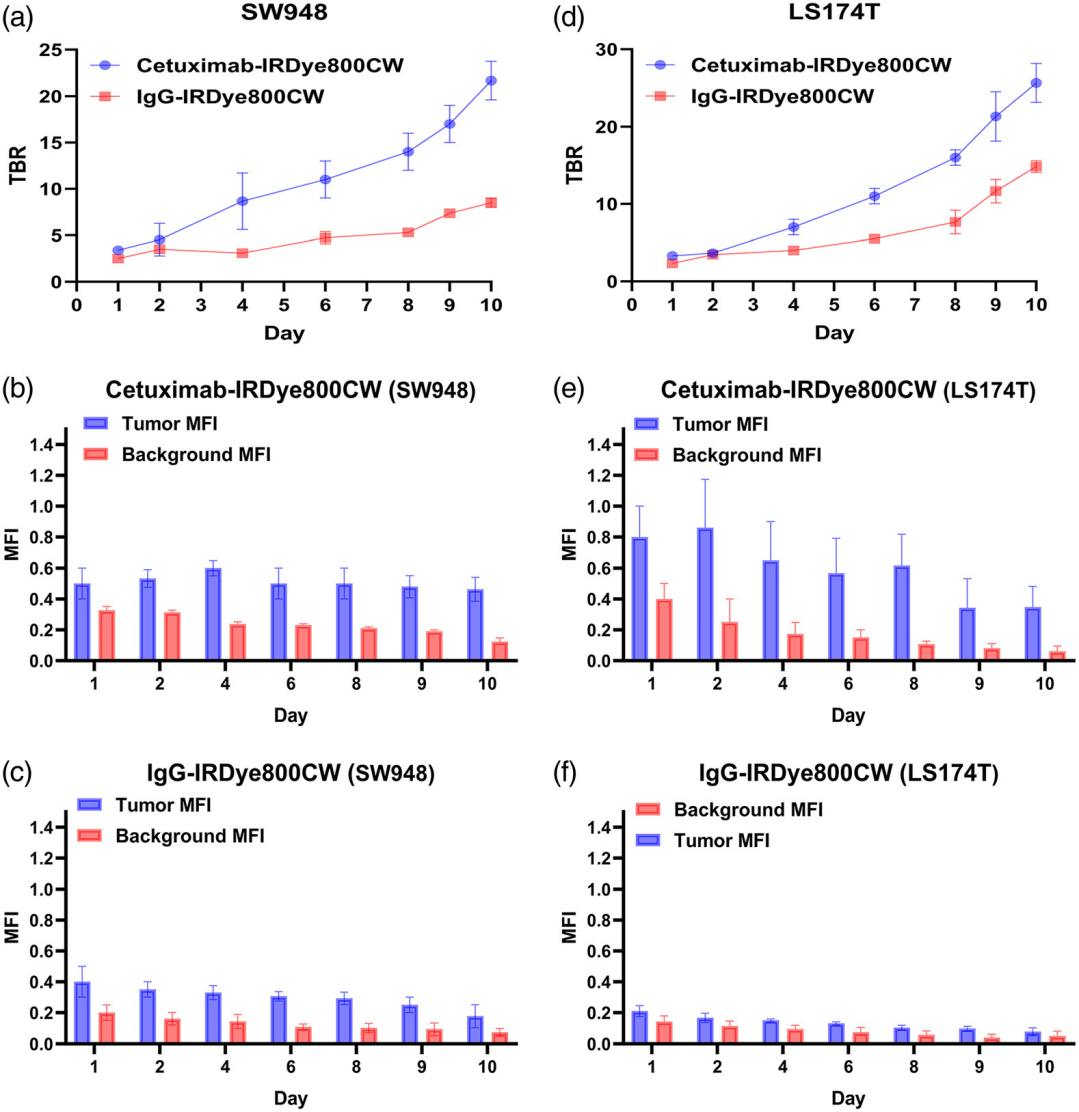
Quantitative analysis of fluorescence imaging in SW948 and LS174T xenograft models [(a)–(c): SW948; (d)–(f): LS174T]. (a) Tumor-to-background ratio (TBR) curves of cetuximab-IRDye800CW and IgG-IRDye800CW groups in SW948 xenografts. The TBR in the cetuximab-IRDye800CW group progressively increased over time, reaching a peak of 21.6±1.71 on day 10. (b) Comparison of mean fluorescence intensity (MFI) between tumor and background regions in SW948 xenografts. Tumor MFI in the cetuximab-IRDye800CW group remained consistently higher than background MFI throughout the imaging period. (c) Temporal changes in background MFI in SW948 xenografts, showing a marked decrease from 0.33 on day 1 to 0.15 on day 10 (p<0.05), indicating progressive clearance of nonspecific signals. (d) TBR curves of LS174T xenografts showing a similar trend, with significantly higher TBR values in the cetuximab-IRDye800CW group than in the IgG-IRDye800CW group at all time points, peaking at 25.6±2.94 on day 10 (p<0.05). (e) Comparison of tumor and background MFI in LS174T xenografts. The cetuximab-IRDye800CW group exhibited stable and strong tumor-specific fluorescence, whereas no significant changes were observed in the control group. (f) Background MFI dynamics in LS174T xenografts, showing a gradual decline from 0.40 on day 1 to 0.05 on day 10 (p<0.05).

In both SW948 and LS174T cell lines, the TBR in the cetuximab-IRDye800CW group progressively increased throughout the 10-day imaging period, reaching a peak on day 10 (SW948: 21.6±1.71; LS174T: 25.6±2.94). These findings indicate that cetuximab-IRDye800CW selectively enhanced tumor fluorescence while reducing background signal, thereby significantly improving fluorescent contrast. Two-way repeated-measures ANOVA showed significant main effects of group and day and a group × day interaction (all p<0.05). Sidak-adjusted post hoc comparisons confirmed that the TBRs of the cetuximab-IRDye800CW group were significantly higher than those of the IgG-IRDye800CW controls at each imaging day [p<0.05, Figs. 2(a) and 2(d)]. MFI analysis, also assessed using two-way repeated-measures ANOVA (group × day) with Sidak-adjusted post hoc tests, demonstrated that tumor MFI in Cetuximab-treated mice consistently exceeded background MFI, whereas background MFI decreased significantly over time (SW948: 0.33 on day 1 versus 0.15 on day 10; LS174T: 0.40 on day 1 versus 0.05 on day 10; all p<0.05), with no significant changes observed in the control group [Figs. 2(b), 2(c), 2(e), and 2(f)]. These findings indicate that cetuximab-IRDye800CW enabled selective tumor-specific fluorescence while maintaining a low background signal, thereby significantly improving overall imaging contrast.

**Fig. 3:**
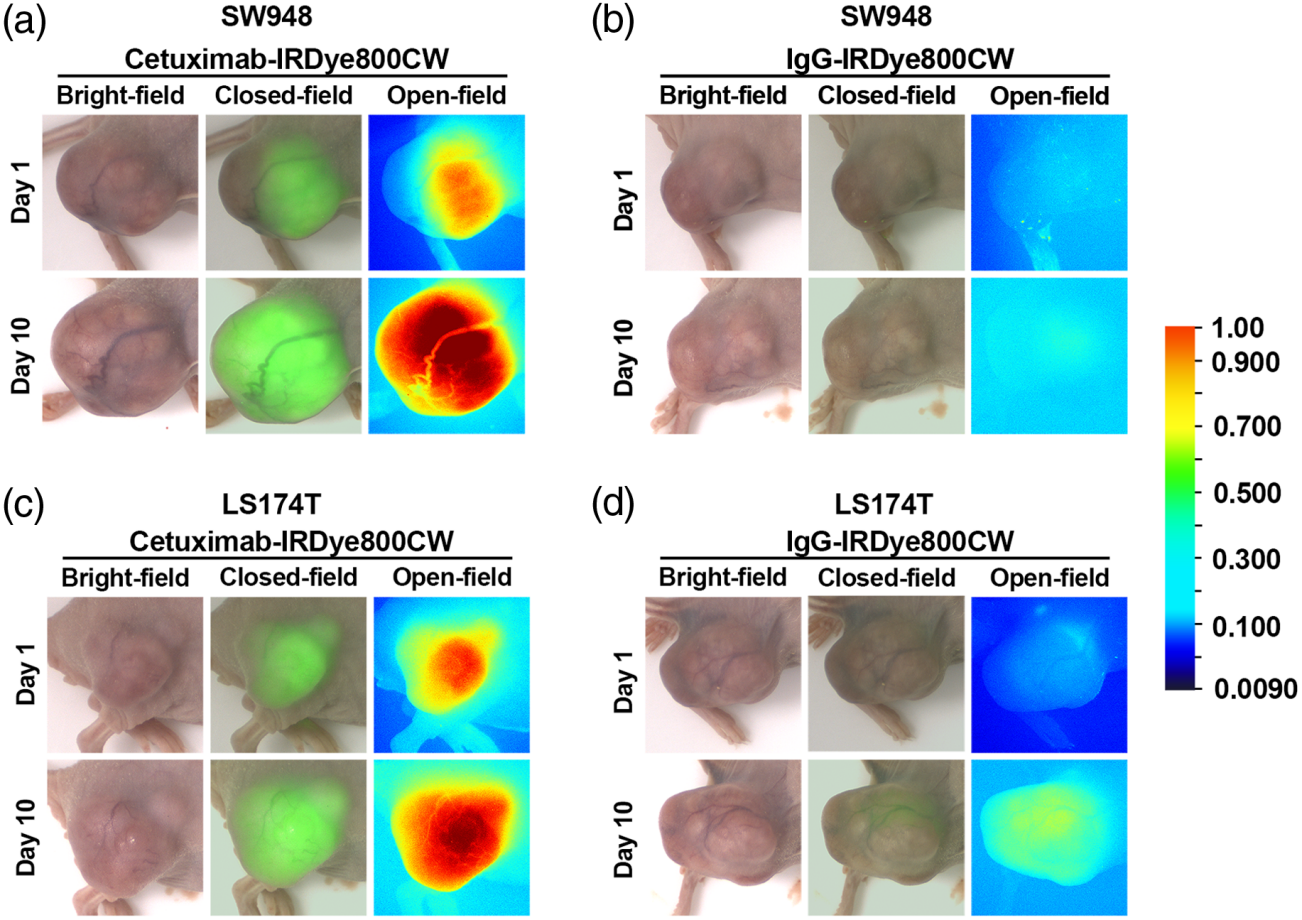
Open- and closed-field fluorescence imaging of SW948 and LS174T xenograft models. (a) Open-field images of SW948 xenografts showing visible tumor fluorescence in the cetuximab-IRDye800CW group from day 1, which intensified by day 10; IgG controls showed only weak background signals. (b) Closed-field images of SW948 xenografts confirming strong, tumor-localized fluorescence in the cetuximab-IRDye800CW group. (c) Open-field images of LS174T xenografts showing progressive tumor-specific fluorescence enhancement in the cetuximab-IRDye800CW group with minimal signal in controls. (d) Closed-field images of LS174T xenografts demonstrating clear, high-contrast fluorescence at tumor sites in the cetuximab-IRDye800CW group compared with negligible background in controls. These results confirm selective, strong, and progressively enhanced tumor-targeted fluorescence with cetuximab-IRDye800CW in both models.

To validate these quantitative results, open- and closed-field fluorescence imaging was performed. In both SW948 and LS174T models, cetuximab-IRDye800CW-treated mice exhibited stronger tumor-specific fluorescent signals compared with controls (Fig. 3). Notably, tumor fluorescence was already visible on day 1 and intensified by day 10, with open-field imaging showing strong red high-intensity signals in the tumor region. By contrast, IgG-treated controls showed only weak background fluorescence without tumor specificity.

**Fig. 4:**
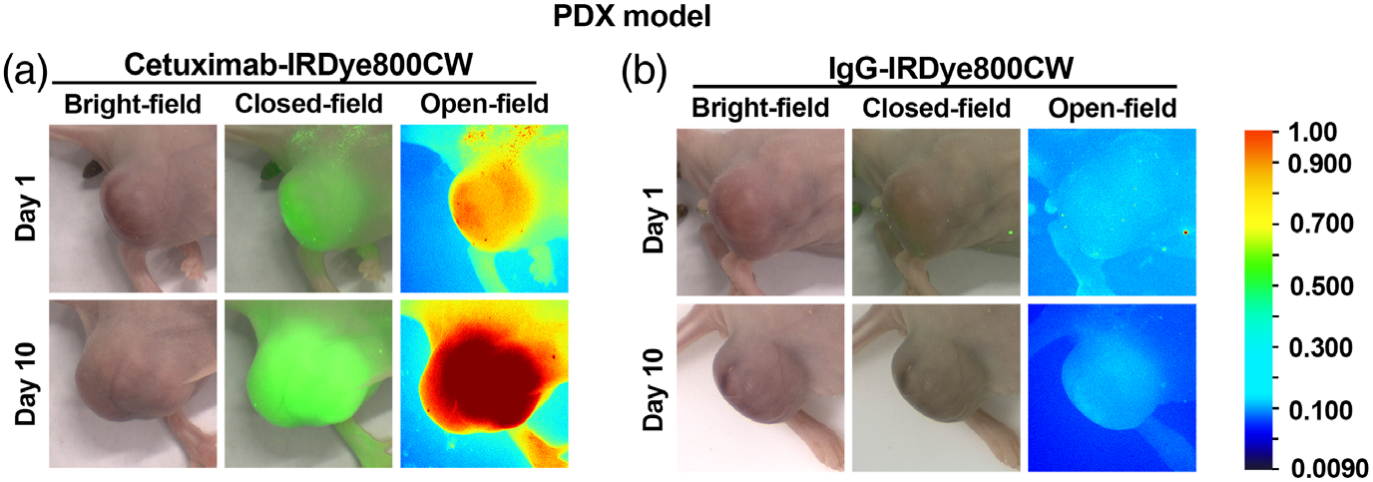
Fluorescence imaging of EGFR-high patient-derived xenograft (PDX) models. (a) Representative fluorescence images on day 1 showing clear tumor-specific signals in the cetuximab-IRDye800CW group, whereas the IgG-IRDye800CW control group exhibited negligible background fluorescence. (b) Fluorescence images on day 10, demonstrating markedly enhanced tumor-localized fluorescence in the cetuximab-IRDye800CW group with no detectable tumor signal in controls. These results confirm stable and progressively increased tumor-targeted fluorescence in the PDX model following cetuximab-IRDye800CW administration.

Consistent results were also observed in the PDX model (Fig. 4). Cetuximab-IRDye800CW-treated mice displayed clear tumor-specific fluorescence from day 1, which further increased by day 10. Conversely, IgG-treated controls showed no appreciable tumor signal across the imaging period. These findings confirm that cetuximab-IRDye800CW provides stable and progressively enhanced tumor-targeted imaging across different models. Although the maximal TBR was observed on day 10, the imaging study was terminated at this time point because the signal intensity had reached a plateau and further tumor growth could interfere with quantitative accuracy and animal welfare. The 10-day observation period was predetermined based on prior pharmacokinetic studies of antibody–dye conjugates, which typically achieve stable tumor retention and background clearance within 7 to 10 days.

**Fig. 5:**
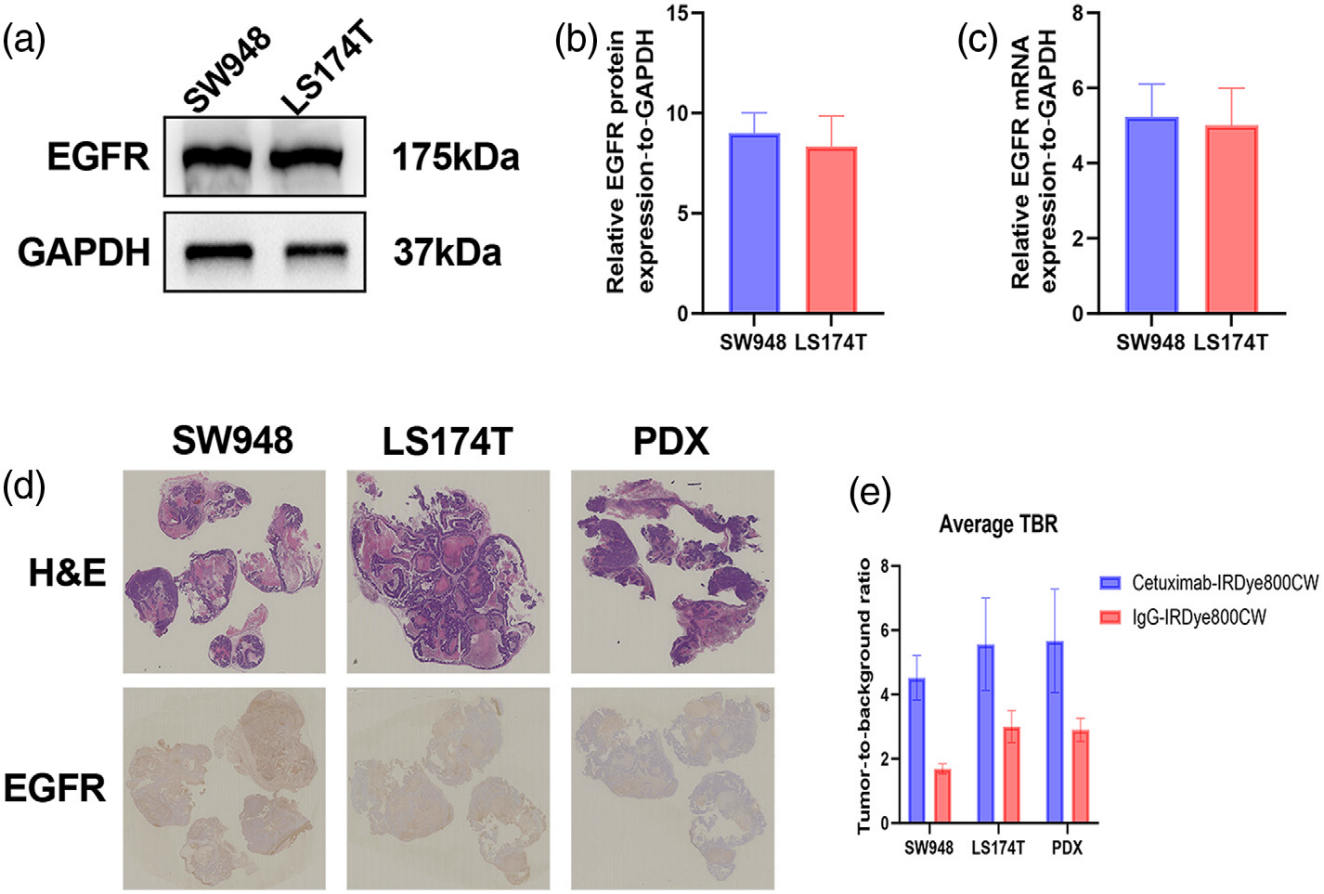
EGFR expression and tumor fluorescence across models. (a) Western blot analysis of EGFR in LS174T and SW948 cell lines. (b) Bar graph depicting EGFR protein levels (densitometry) in LS174T and SW948. (c) Bar graph showing EGFR mRNA expression in LS174T and SW948 (RT-qPCR). (d) H&E staining and EGFR immunohistochemistry of tumors in LS174T, SW948, and PDX models after euthanasia, showing strong membrane EGFR expression. (e) Tumor-to-background ratio (TBR) comparison demonstrating significantly higher values in cetuximab-IRDye800CW-treated mice compared with IgG controls across all three models.

To further explore the molecular basis of this enhanced fluorescent contrast, EGFR expression levels were examined. Western blot and reverse transcription quantitative polymerase chain reaction (RT-qPCR) analyses confirmed high levels of EGFR protein and mRNA in two colorectal cancer cell lines (LS174T and SW948) [Figs. 5(a)–5(c)]. Consistently, immunohistochemistry revealed strong EGFR staining not only in both cell lines but also in a PDX model [Fig. 5(d)]. In line with these findings, cetuximab-IRDye800CW produced markedly enhanced fluorescent signals across all three EGFR-overexpressing models, as reflected by significantly elevated TBRs compared with IgG controls [SW948: 4.52±1.01 versus 1.68±0.22; LS174T: 5.57±1.86 versus 3.00±0.58; and PDX: 5.66±1.54 versus 2.90±0.36; all p<0.05, Fig. 5(e)]. Collectively, these results demonstrate that cetuximab-IRDye800CW generates strong and tumor-specific fluorescence in multiple EGFR-high models, thereby confirming its ability to target EGFR *in vivo*.

## 4 Discussion

Although cetuximab-IRDye800CW has indeed been explored clinically for head and neck, brain, and pancreatic cancers, its translation to colorectal surgery remains at an early preclinical stage. The present study provides tumor-type-specific evidence supporting its feasibility in CRC, addressing unique anatomical and biological features distinct from previously tested tumors. Unlike previous reports focusing on head-and-neck or brain tumors, our work represents one of the few systematic evaluations of cetuximab-IRDye800CW in colorectal cancer. By combining cell-line-derived and PDX models with longitudinal dual-mode imaging, this study comprehensively assessed the potential of cetuximab-IRDye800CW in CRC. The probe demonstrated a progressive increase in TBR, reaching a peak on day 10, accompanied by a marked decline in background MFI. These findings suggest that imaging specificity is not solely dependent on tumor uptake but also closely linked to background clearance kinetics. This extends previous reports showing peak signals for antibody-based IRDye800CW probes at 48 to 96 h,^8^^,^^12^ indicating that in long-term dynamic imaging, background clearance may further enhance TBR at later time points and provide new insights for optimizing the dosing-to-surgery window in clinical translation.

In this study, the imaging characteristics of cetuximab-IRDye800CW were systematically validated in two well-established EGFR-overexpressing colorectal cancer cell lines (LS174T and SW948) as well as in an EGFR-high PDX model. The probe consistently generated strong and stable fluorescence across these models, with TBRs significantly higher than those of IgG controls, thereby confirming its specific targeting capability for EGFR. This finding is consistent with previous reports indicating that receptor density is a critical determinant of probe uptake and imaging contrast.^13^ Notably, the IgG-IRDye800CW control also generated low-level fluorescence, consistent with the enhanced permeability and retention (EPR) effect that leads to nonspecific macromolecule accumulation.^14^^,^^15^ In addition, the gradual decline in background MFI confirmed that the signal was predominantly tumor-specific rather than nonspecific retention. It is also important to note that receptor expression and imaging outcomes are not strictly linear; tumor perfusion, stromal barriers, and hypoxia/necrosis may influence probe distribution, explaining inter-model variability.^16^ Clinically, EGFR overexpression is observed in ∼60% to 80% of colorectal cancers,^17^^,^^18^ with a tendency toward higher prevalence in left-sided and metastatic lesions.^19^^,^^20^ This widespread expression supports the clinical relevance of using EGFR-targeted probes such as cetuximab-IRDye800CW for intraoperative fluorescence imaging in CRC.

In CRC fluorescence-guided surgery, the carcinoembryonic antigen (CEA)-targeted probe SGM-101 has advanced to multicenter clinical trials, showing that a 10 mg dose administered 4 days before surgery significantly improved sensitivity and lesion detection.^21^^,^^22^ Our findings suggest that EGFR-targeted imaging provides comparable contrast and specificity, particularly in tumors with high EGFR expression, making it a valuable complement to CEA-targeted strategies. Compared with nonspecific agents such as ICG, cetuximab-IRDye800CW provides superior tumor specificity and reduced background interference. In addition, the IRDye800CW fluorophore enables deeper tissue penetration owing to its near-infrared emission wavelength and reduced light scattering.^23^^–^^25^ These features may prove especially beneficial in anatomically complex CRC cases or those with minimal residual disease. Moreover, the consistency observed between open-field and closed-field imaging parallels current clinical workflows, which combine intraoperative navigation with specimen margin assessment.

Importantly, beyond macroscopic tumor visualization, our study also included a preliminary assessment of detection sensitivity. Small reimplanted tumor fragments (∼1  mg) within the wound bed remained visible under both open- and closed-field imaging conditions, suggesting that cetuximab-IRDye800CW may enable intraoperative identification of minimal residual disease. Although exploratory, this finding supports the feasibility of applying cetuximab-IRDye800CW for intraoperative margin detection and real-time surgical decision-making during colorectal cancer resection. Consistent with our observation, recent clinical studies in other tumor types have demonstrated that antibody–dye conjugates such as cetuximab-IRDye800CW can delineate tumor-positive or close margins with high sensitivity and negative predictive value, further confirming their potential for intraoperative margin assessment.^6^^,^^24^^,^^26^ In anatomically complex colorectal procedures—particularly in low rectal or mesorectal regions, where achieving negative circumferential margins is technically challenging—fluorescence-guided margin evaluation could help reduce positive-margin rates and minimize the need for re-excision or adjuvant therapy. Early-phase clinical data from other epithelial malignancies, including penile squamous cell carcinoma, also support the safety and feasibility of EGFR-targeted probes for intraoperative use.^27^ Collectively, these findings highlight a promising translational direction for cetuximab-IRDye800CW imaging toward real-time surgical guidance and improved local oncologic control in colorectal cancer.

This study has several limitations, including a relatively small sample size and the lack of systematic comparisons of different doses and time windows. In addition, EGFR heterogeneity may limit probe uptake in certain tumors, and pharmacokinetics and safety profiles were not assessed. Future research should focus on: (1) optimizing dosing and imaging schedules to define the ideal clinical window for CRC; (2) validating EGFR-based stratification in larger PDX cohorts and clinical trials, and directly comparing EGFR-IRDye800CW with CEA-SGM-101; (3) integrating artificial intelligence-based algorithms to improve the objectivity and real-time interpretation of intraoperative imaging; and (4) conducting systematic pharmacokinetic, safety, and long-term oncologic outcome studies to support clinical translation.

## 5 Conclusion

In conclusion, our study demonstrates that cetuximab-IRDye800CW enables stable, tumor-specific, and high-contrast fluorescence imaging in colorectal cancer models, with performance closely linked to EGFR expression. These findings not only validate the probe’s potential to improve intraoperative tumor detection and margin assessment but also highlight its translational relevance as a complementary strategy to existing fluorescence agents. With further optimization of dosing regimens, patient stratification, and clinical validation, cetuximab-IRDye800CW may become an important tool for enhancing surgical precision and improving long-term oncologic outcomes in colorectal cancer.

## Data Availability

The datasets analyzed in this study are available from the corresponding author upon justified request.
